# Reassessing the Risk: A Retrospective Analysis of CLABSI Risk in Femoral, Internal Jugular, and Subclavian Central Venous Catheters

**DOI:** 10.1155/ccrp/8193419

**Published:** 2025-04-29

**Authors:** Alexandra Vaughan-Masamitsu, Wesley Paulson, Robert Hodes, Cain Dudek

**Affiliations:** ^1^Penn State College of Medicine, Pennsylvania State University, Hershey, Pennsylvania, USA; ^2^St. George's School of Medicine, St. George's University, University Centre Grenada, West Indies, Grenada

**Keywords:** central line–associated bloodstream infection, central venous catheter, CLABSI, femoral vein, infection risk, internal jugular vein, retrospective cohort study, subclavian vein, TriNetX

## Abstract

**Background:** Central line–associated bloodstream infections (CLABSIs) represent a significant healthcare challenge due to their association with increased morbidity, mortality, and financial burden. Current guidelines discourage the use of the femoral vein (FV) for central venous catheter (CVC) placement due to a perceived higher infection risk compared to the internal jugular vein (IJV) or subclavian (SCV) sites. However, recent evidence questions this assumption and suggests that femoral CVCs may carry similar risks to other sites, emphasizing the need for updated analyses.

**Objective:** The goal of this study was to address the misconception that femoral CVCs have a higher associated risk for developing CLABSI compared to other central line sites. This study evaluates risk for CLABSI across FV, IJV, and SCV sites.

**Methods:** Using the TriNetX Research Network to conduct a retrospective cohort analysis, initial queries identified 99,216 patients who were encountered between 2014 and 2025 for CVC placement. Following propensity score matching, 65,265 of these patients were retained for statistical analysis. Patients were categorized based on anatomic CVC placement sites into IJV, SCV, and FV cohorts. CLABSI incidence was determined using ICD-10-CM codes within 1 day to 1 month post-CVC insertion. Sensitivity analyses were conducted for the 2014–2025 period, as well as for the 2014–2019 and 2019–2025 periods to assess overall risk and evaluate for changes in CLABSI risk by anatomic site over time. Outcomes were compared using risk percentages, risk ratios, and odds ratios with 95% confidence intervals to compare differences in risk for CLABSI across different sites.

**Results:** Overall, femoral CVCs were not associated with a statistically significant higher risk of CLABSI compared to IJV or FV CVCs from the overall period of 2014–2025. Only the risk difference between IJV and SCV CVCs over 2014-2025 showed a statistically significant difference. IJV CVCs were associated with a higher risk of CLABSI compared with SCV CVCs, with a risk difference of 0.089% (95% CI: 0.006%, 0.171%, *Z* = 2.11, *p*=0.0348), a risk ratio of 1.708 (95% CI: 1.033, 2.826), and an odds ratio of 1.71 (95% CI:1.033, 2.831). Over the 2014–2019 period, there was no statistically significant risk difference between the IJV and FV cohorts (risk difference 0.09%, 95% CI: −0.035%, 0.215%, *Z* = 1.415, *p*=0.1569). Comparing the IJV to SCV CLABSI rates for the 2014–2019 period, the risk difference was 0.112% (95% CI: −0.009%, 0.234%, *Z* = 1.81, *p*=0.07). For the 2019–2025 period between the IJV and FV cohorts, the risk difference was −0.077% (higher risk in the FV cohort), which was not a statistically significant difference (95% CI: −0.193%, 0.04%, *Z* = −1.289, *p*=0.1974). Comparing the IJV to SCV CLABSI rates for the 2019–2025 period, the risk difference was 0.117% (95% CI: = −0.006%, 0.24%, *Z* = 1.861, *p*=0.0627), which was not a statistically significant difference.

**Conclusions:** This study challenges the prevailing assumption that femoral CVCs carry a higher risk of CLABSI compared to IJV and SCV sites, showing no significant difference in risk. These findings suggest that avoidance of the FV for CVC placement out of concern for infection may unnecessarily limit clinical options without improving patient outcomes. Emphasizing site-specific risks like technical complications and anatomical considerations over infection concerns could simplify decision-making and enhance personalized care in CVC placement.

## 1. Introduction

In August 2007, the Centers for Medicare and Medicaid Services (CMS) announced that Medicare would no longer reimburse healthcare institutions for various preventable healthcare-associated events [1]. Among these are hospital-acquired infections such as central line–associated bloodstream infections (CLABSIs), which occur at a rate of 0.87 per 1000 central line days in the U.S. intensive care units (ICUs) [2]. CLABSIs are a significant cause of morbidity and mortality, with an associated mortality rate of 12%–15% [3]. In addition to the negative patient outcomes, CLABSIs increase the length of hospitalization and have financial ramifications due to Medicare's lack of reimbursement. In this setting, it is vital for healthcare systems and providers to have an accurate understanding of the risk of CLABSI.

Within the past two decades, the term catheter-related bloodstream infection (CRBSI) emerged as a clinical definition of bloodstream infections attributed to intravascular catheters. The Centers for Disease Control (CDC) defines a CRBSI as a clinical diagnosis which more accurately identifies the catheter as the infectious source through specific microbiologic testing of the catheter. Notably, this can be problematic because catheters are not always removed, and the catheters are not always sent for laboratory testing. Additionally, both CLABSI and CRBSI use the same ICD-10 code (T80.211) for diagnosis. As such, this paper uses the term CLABSI to refer to any bloodstream infection attributable to central venous catheters (CVCs) with or without microbiological analysis of the suspected catheter. Guidelines from 2011 recommend avoiding femoral venous access in adult patients (1A recommendation), as the femoral site is believed to harbor a higher microbial burden due to its proximity to the groin [4]. This has led to a preference for internal jugular vein (IJV) or subclavian CVCs, despite their own associated risks, such as pneumothorax or carotid artery injury [5].

Recent evidence, however, challenges the assumption that femoral CVCs are more prone to CLABSI. Studies suggest that when proper insertion techniques and infection control measures are employed, the CLABSI rates for femoral CVCs may be comparable to those for internal jugular or subclavian lines. A systematic review and meta-analysis by Marik et al. revealed a shift over time: Older studies indicated a lower risk of CLABSI with internal jugular access, whereas newer studies show no significant difference in CLABSI rates across different sites [6]. A more recent study published in 2025 and based on clinical data in French ICUs from 2018 to 2022 found no significant difference in CLABSI rates between the three insertion sites [7]. A third study based on a retrospective cohort from 2016 to 2018 from one hospital system in Greece found a higher risk of CLABSI in the femoral vein (FV) site, yet this difference was not statistically significant [8]. These findings question the prevailing notion that the femoral site is inherently riskier regarding infection risk, advocating for a reassessment of femoral CVC placement in certain clinical contexts where other sites may pose technical challenges or additional risks.

Conversely, the study by Parienti et al. in 2015 examined major catheter-related complications, utilizing a composite endpoint of CLABSI and symptomatic deep vein thrombosis (DVT) [9]. While this study reported a higher risk of composite endpoint with femoral CVCs compared to subclavian and internal jugular sites, its findings must be interpreted with caution. Patients with femoral catheters inherently carry a higher baseline risk for DVT due to being nonambulatory, a confounding factor that may have influenced the results. The inclusion of symptomatic DVT as part of the composite endpoint complicates direct comparisons of CLABSI risk across insertion sites. Further breakdown of the composite endpoint reveals 1.4% of patients in the IJV cohort (*N* = 845) developed a bloodstream infection, while 1.2% of patients in the FV cohort (*N* = 844) had the same outcome [9].

These contradictory findings question the prevailing notion that the femoral site is at a higher risk of CLABSI, advocating for a reassessment of femoral CVC placement in certain clinical contexts where other sites may pose technical challenges or additional risks.

This study compares the incidence of bloodstream infection among patients with CVCs placed in three anatomical sites: IJV, subclavian vein (SCV), and FV. The primary goal is to address differences in recent research regarding CLABSI rates with FV CVCs. The CDC guidelines from 2011 regarding CVC placement rested on data that FV CVCs have a higher risk of CLABSI compared to other CVC sites [4]. This has been called into question by prior studies [6, 7]. By evaluating CLABSI rates across various central line locations, this research seeks to further investigate whether femoral CVCs do indeed have a higher risk of CLABSI.

## 2. Methods

### 2.1. Study Design

This retrospective cohort analysis utilized data collected from the Global Collaborative Network with Natural Language Processing (NLP), incorporating information from 132 healthcare organizations (HCOs) within the TriNetX Research Network. The data were gathered from TriNetX on February 12, 2025, and were used to evaluate the incidence of CLABSIs in patients who had CVCs placed in one of three anatomical sites: the IJV, FV, or SCV. A primary analysis was conducted to assess cohorts between 2014 and 2025 to assess general CLABSI rates associated with each anatomic location. As mentioned in the Introduction, some data indicated infection rates changed over time. To this end, a separate sensitivity analysis was conducted using the time periods of 2014–2019 and 2019–2025, resulting in an additional six cohorts: patients with IJV lines placed from 2014 to 2019 and 2019 to 2025, patients with FV lines placed from 2014 to 2019 and 2019 to 2025, and patients with SCV lines placed from 2014 to 2019 and 2019 to 2025.

The primary outcome of interest was the development of bloodstream infection due to CVC within a time window of 2 days to 1 month following CVC insertion. This outcome was identified by the ICD-10 codes T80.211 and T80.211A, as outlined in Table 1. Inclusion criteria required patients to be between 18 and 90 years old who had received a CVC in either the IJV, FV, or SCV while hospitalized. To control confounding variables, patients were excluded if they had a diagnosis of sepsis or burns involving 10%–99% body surface area within 31 days before hospital admission. Furthermore, patients were excluded if they required multiple CVCs, hemodialysis, or percutaneous mechanical circulatory support devices. Inclusion and exclusion factors were selected using specific ICD and CPT codes, as outlined in Tables 2 and 3.

### 2.2. Study Population and Demographics

A total of 99,216 patients with a CVC placed in one of the three specified anatomic locations were included in the study, with 65,265 patients retained for initial statistical analyses following propensity score matching. Propensity score matching was employed to minimize confounding factors between the cohorts. Matching was based on patient demographics including age at the time of the index event, sex, ethnicity, race, comorbidities, and various laboratory values. Balancing for mechanical ventilation, vasopressor (norepinephrine and epinephrine), and lactate was performed to provide objective data regarding illness severity. Diagnoses of neoplasms (any), chronic kidney disease (not on hemodialysis), and diabetes mellitus, along with glucocorticoid use, neutrophil count, and hemoglobin A1c were balanced to provide insight on baseline risk for infection between cohorts. A 1:1 matching ratio was used to balance these variables across the different CVC sites and time periods, allowing for a direct comparison of CLABSI rates. In each comparative analysis, a small number of patients were excluded due to the outcome of interest occurring outside of the time window of the study. Patient demographics for each cohort prior to and following propensity score matching can be found in Tables 4, 5, 6, and 7.

### 2.3. Statistical Analysis

Following propensity score matching, the cohorts were compared based on their CLABSI rates. The risk percent for CLABSI was calculated for each cohort, and risk differences, risk ratios, and odds ratios were derived to quantify the comparative risk for developing CLABSI between different CVC placement sites. Risk differences were presented with 95% confidence intervals (CI), and significance was assessed using *Z* tests for differences in proportions. A *p* value < 0.05 was considered statistically significant. The risk ratio and odds ratio were also computed with 95% CI for each comparison. All statistical analyses were performed using the TriNetX analytics platform.

## 3. Results

### 3.1. IJV vs. FV Comparative Analysis: 2014–2025

The initial IJV cohort contained 53,350 participants, while the FV cohort included 24,214. After matching, both groups were reduced to 23,073 participants.

The risk analysis for the IJV and FV cohorts showed that the IJV group had a risk percentage of 0.169%, while the FV group exhibited a risk of 0.225%. The risk difference between IJV and FV was 0.056%, with a 95% CI ranging from −0.137% to 0.025% (*Z* = −1.364, *p*=0.1725), indicating no statistically significant difference in risk between these two groups. The risk ratio of IJV versus FV was 0.75 (95% CI: 0.495, 1.136), and the odds ratio was 0.75 (95% CI: 0.495, 1.136), also suggesting no significant difference between the IJV and FV cohorts.

### 3.2. IJV vs. SCV Comparative Analysis: 2014–2025

The initial cohort sizes for the IJV and SCV groups from 2014 to 2025 were 53,350 and 19,449 patients, respectively. After propensity score matching, both the IJV cohort and the SCV cohort retained 19,119 patients for the final analysis.

In a risk analysis, the risk percentage in the IJV group was 0.214% compared to 0.126% in the SCV group. The difference in risk between the IJV and SCV groups was calculated at 0.089%, with a 95% CI of 0.006%–0.171% (*Z* = 2.11, *p*=0.0348), indicating a statistically significant difference with higher risk in the IJV cohort. Additionally, the risk ratio for IJV versus SCV was found to be 1.708 (95% CI: 1.033, 2.826), and the odds ratio was 1.71 (95% CI: 1.033, 2.831), both of which supported a higher likelihood of the outcome in the IJV group.

### 3.3. IJV vs. FV Comparative Analysis: 2014–2019 and 2019–2025

In the 2014–2019 period, 8881 patients with IJV catheters and 8881 patients with FV catheters were included in the analysis following propensity score matching. The CLABSI rate in the IJV cohort was 0.225%, while in the FV cohort it was 0.135%. The risk difference between the two cohorts was 0.09% (95% CI: −0.035%, 0.215%, *Z* = 1.415, *p*=0.1569). The risk ratio for CLABSI in the IJV versus FV cohort was 1.667 (95% CI: 0.815, 3.407), and the odds ratio was 1.668 (95% CI: 0.815, 3.414).

For the 2019–2025 period, 14,344 patients with IJV lines and 14,344 patients with FV lines were included after matching. The risk of CLABSI in the IJV cohort was 0.216%, compared to 0.293% in the FV cohort, with a risk difference of −0.077% (95% CI: −0.193%, 0.04%, *Z* = −1.289, *p*=0.1974). The risk ratio was 0.738 (95% CI: 0.464, 1.173), and the odds ratio was 0.738 (95% CI: 0.463, 1.174).

These data derived from sensitivity analyses demonstrate no statistically significant difference in bloodstream infection risk existed between the two cohorts regardless of the time period. While these data are consistent with recent literature questioning infection risk, it conflicts with the notion that rates of infection have changed over time. The incidence of infection is higher in the FV 2014–2025 cohort compared to the 2014–2019 cohort. This may be partially explained by the large difference in the number of patients receiving femoral central catheters in the 2019–2025 group.

### 3.4. IJV vs. SCV Comparative Analysis: 2014–2019 and 2019–2025

When comparing IJV to SCV CLABSI rates in the 2014–2019 period, 9811 patients from each cohort were analyzed after matching. The CLABSI rate in the IJV cohort was 0.245%, while in the SCV cohort it was 0.133%. The risk difference was 0.112% (95% CI: −0.009%, 0.234%, *Z* = 1.81, *p*=0.07). The risk ratio for CLABSI in IJV versus SCV was 1.846 (95% CI: 0.941, 3.624), and the odds ratio was 1.848 (95% CI: 0.941, 3.632).

For the 2019–2025 period, 9398 patients from each cohort were included after matching. The CLABSI rate in the IJV cohort was 0.245%, compared to 0.128% in the SCV cohort. The risk difference between the two groups was 0.117% (95% CI: −0.006%, 0.24%, *Z* = 1.861, *p*=0.0627), with a risk ratio of 1.917 (95% CI: 0.954, 3.85) and an odds ratio of 1.919 (95% CI: 0.954, 3.859).

## 4. Discussion

CLABSIs represent a significant cause of morbidity and mortality in hospitalized patients, imposing both clinical and financial burdens [2, 3]. Utilizing the largest single-study retrospective cohort to date, this study aimed to investigate whether there is a difference in CLABSI risk based on placement location in the FV, IJV, and SCV.

### 4.1. Principal Findings

Our study revealed no statistically significant risk difference between the IJV and FV cohorts from 2014 to 2025. There was a statistically significant risk difference when comparing the IJV to SCV cohorts from 2014 to 2025, with the higher risk of CLABSI in the IJV group. In the time-period sensitivity analyses, we found no statistically significant difference between CLABSI rates when comparing the FV to IJV cohorts in 2014–2019 and 2019–2025. Interestingly, both time-period sensitivity analyses comparing SCV to IJV cohorts demonstrate no statistically significant difference in risk for bloodstream infection while the primary analysis showed a statistically significant difference. However, the sensitivity analyses were quite close to approaching statistical significance which when combined, likely increased the power of the study to detect a difference between the two cohorts.

### 4.2. Clinical Implications

The present findings suggest that from 2014 to 2025, there has not been a higher risk of bloodstream infection from CVCs when placed in either the FV or IJV. The 2014–2019 IJV and FV cohort did not have a statistically significant difference in risk between the IJV group and the FV group. This is consistent with the meta-analysis and systematic review by Marik et al. in finding that there is not a higher risk of CLABSI in the FV site than in the IJV site [6].

Our findings suggest CVCs inserted into the FV do not carry a higher risk of CLABSI when compared with catheters inserted into the IJV. This is clinically meaningful because it suggests that hospital-specific guidelines discouraging the use of femoral CVCs specifically for infection concerns should be reassessed.

Stringent infection control measures should be in place for insertion and maintenance of all central lines, and the option to use FV CVCs should not be limited by a concern of higher risk of CLABSI compared to IJV CVCs.

This could simplify decision-making when determining a route for CVC access, and clinicians could focus primarily on the risks of other site-based complications such as carotid artery injury or pneumothorax, rather than having to combine these complications with perceived infection risk.

### 4.3. Limitations

There are several limitations to this study that must be considered when interpreting the results. First, as a retrospective cohort study, our analysis is subject to selection bias. The reliance on ICD codes for identifying patients and complications may be prone to misclassification errors. We did not have access to detailed clinical data on the insertion techniques or infection control measures used at individual healthcare facilities, which may have influenced the outcomes. Although the cohorts were balanced by mechanical ventilation, the need for vasopressors, and lactate, these serve only as a surrogate to illness severity. The duration of catheterization, when in the hospital course the catheterization occurred, or time from insertion to infection were not accounted for. There could be significant heterogeneity between patients within each group. We addressed this limitation by using propensity score matching, with the aim to reduce differences in demographics and clinical status among the groups. Propensity score matching also allowed both groups within each cohort to be of a similar size.

Next, different institutions may utilize varying ICD codes for CLABSI, potentially leading to missed cases in our final analysis. Finally, the time covered by this review spans more than a decade, during which practices and guidelines related to CVC insertion and infection control have evolved. To try to account for changes in practices, CLABSI risk was assessed in each group in two separate time periods, 2014–2019 and 2019–2025.

### 4.4. Future Studies

Given that the recent data suggest that with modern infection control methods of CVC placement, there is not a statistically significant difference in risk of CLABSI in the FV cohort compared to the IJV cohort, we suggest a reappraisal of the current guidelines recommending avoiding femoral venous access. Various studies have found conflicting results regarding CLABSI risk based on location [2, 6, 7, 10]. Marik et al. performed a systematic review in 2012 and found similar rates of CLABSI development between the femoral, subclavian, and internal jugular sites [6]. However, later studies including by Toor et al. conflict with this, which found that the femoral site had a higher incidence of CLABSI compared with the internal jugular and subclavian sites [2]. A recent study based on data from ICUs in France from 2018 to 2022 found no significant difference in CLABSI risk based on location [7]. Given that systematic reviews are an essential component of clinical guideline development, and that there are more studies since the Marik et al. paper, a systematic review of the rate of CLABSI based on catheter placement would be the next step toward a reappraisal of the CVC guidelines discouraging femoral access due to infectious concerns [11].

## 5. Conclusion

The present study provides additional evidence that from 2014 to 2025, femoral CVCs are not associated with a higher risk of CLABSI when compared to internal jugular CVCs. These findings challenge the current clinical preference for avoiding the femoral site and highlight the need for revisiting the guidelines to reflect more recent evidence regarding CLABSI.

## Figures and Tables

**Table 1:** ICD-10 codes for outcome definition.

Code	Outcome definition
ICD-10-CM T80.211	Bloodstream infection due to central venous catheter
ICD-10-CM T80.211A	Bloodstream infection due to central venous catheter, initial encounter

**Table 2:** ICD-10 codes for inclusion criteria.

Code	Inclusion criteria
ICD-10-PCS 05HM33Z	Insertion of infusion device into right internal jugular vein, percutaneous approach
ICD-10-PCS 05HN33Z	Insertion of infusion device into left internal jugular vein, percutaneous approach
ICD-10-PCS 05H533Z	Insertion of infusion device into right subclavian vein, percutaneous approach
ICD-10-PCS 05H633Z	Insertion of infusion device into left subclavian vein, percutaneous approach
ICD-10-PCS 06HM33Z	Insertion of infusion device into right femoral vein, percutaneous approach
ICD-10-PCS 06HN33Z	Insertion of infusion device into left femoral vein, percutaneous approach

**Table 3:** ICD-10 codes for exclusion criteria.

Code	Exclusion criteria
ICD-9-CM 39.95	Hemodialysis
ICD-10-CM A41.9	Sepsis, unspecified organism
ICD-10-CM T31.1-T31.9	Burns involving 10%–90% of body surface
CPT 33946	Extracorporeal membrane oxygenation (ECMO)/extracorporal life support (ECLS) provided by physician; initiation, veno-venous
CPT 33947	Extracorporeal membrane oxygenation (ECMO)/extracorporal life support (ECLS) provided by physician; initiation, veno-arterial
CPT 33948	Extracorporeal membrane oxygenation (ECMO)/extracorporal life support (ECLS) provided by physician; daily management, each day, veno-venous
CPT 33949	Extracorporeal membrane oxygenation (ECMO)/extracorporal life support (ECLS) provided by physician; daily management, each day, veno-arterial
CPT 33952	Extracorporeal membrane oxygenation (ECMO)/extracorporal life support (ECLS) provided by physician; insertion of peripheral (arterial and/or venous) cannula(e), percutaneous, 6 years or older (includes fluoroscopic guidance, when performed)
CPT 33990	Insertion of ventricular assist device, percutaneous, including radiological supervision and interpretation; left heart, arterial access only
CPT 33991	Insertion of ventricular assist device, percutaneous, including radiological supervision and interpretation, left heart, both arterial and venous access, with transseptal puncture
CPT 33995	Insertion of ventricular assist device, percutaneous, including radiological supervision and interpretation; right heart, venous access only
CPT 36569	Insertion of peripherally inserted central venous access device, without subcutaneous port, without imaging guidance; age 5 years or older
CPT 36571	Insertion of peripherally inserted central venous access device, with subcutaneous port; age 5 years or older

**Table 4:** Patient demographics for IJV vs FV analysis between 2014 and 2025.

**Cohort demographics before and after matching** **Comparison 1: Internal jugular vein vs. femoral vein**				
	**Patients with IJV CVC** **Before matching = 53,530** **After matching = 23,073**		**Patients with FV CVC** **Before matching = 24,214** **After matching = 23,073**	
**Demographic**	**Before matching**	**After matching**	**Before matching**	**After matching**

Age at index				
Age (in years)	Mean ± SD 61.8 ± 16.3 *N* = 53,350	Mean ± SD 60.4 ± 17.0 *N* = 23,073	Mean ± SD 59.8 ± 18.1 *N* = 24,214	Mean ± SD 60.1 ± 17.9 *N* = 23,073
Sex				
Female	23,398	9778	10,138	9751
Male	28,486	12,608	13,338	12,628
Race and ethnicity				
Unknown race	7064	3515	3789	3591
Unknown ethnicity	12,277	5584	5801	5574
White	29,716	11,906	12,266	11,814
Black or African American	9906	5499	5693	5314
Asian	4142	908	1103	1092
Other race	2093	956	1004	955
Hispanic or Latino	5499	1976	2031	1968
Not Hispanic or Latino	35,574	15,513	16,382	15,531

**Table 5:** Patient characteristics for IJV vs FV analysis between 2014 and 2025.

**Cohort characteristics before and after matching** **Comparison 1: Internal jugular vein vs. femoral vein**				
	**Patients with IJ CVC** **Before matching = 53,530** **After matching = 23,073**		**Patients with FV CVC** **Before matching = 24,214** **After matching = 23,073**	
**Characteristic**	**Before matching**	**After matching**	**Before matching**	**After matching**

Diagnoses				
Diabetes mellitus (DM)	14,793	5820	5982	5773
Neoplasms	9026	2261	2496	2469
Chronic kidney disease (CKD)	16,247	6013	6235	6068
Procedure from 7 days prior to same day as index event				
Respiratory ventilation: Less than 24 h	2833	2553	3242	2689
Respiratory ventilation: 24–96 consecutive hours	5538	4127	4435	4031
Respiratory ventilation: Greater than 96 consecutive hours	5553	3740	3912	3697
Introduction of nutritional substance into central vein, percutaneous approach	556	119	126	125
Medications administered from 7 days prior to same day as index event				
Glucocorticoids	20,574	8378	8780	8447
Epinephrine	923	382	407	389
Norepinephrine	11,001	5202	5992	5473
Labs				
Lactate (mmol/L)	Mean ± SD 2.3 ± 2.6 *N* = 16,535	Mean ± SD 2.8 ± 3.2 *N* = 9295	Mean ± SD 3.5 ± 3.8 *N* = 10,844	Mean ± SD 3.3 ± 3.7 *N* = 9908
Lactate: 0–2 mmol/L	12,105	6096	6194	5891
Lactate: 2–4 mmol/L	7540	4758	5226	4746
Lactate: > 4 mmol/L	5023	4164	5167	4339
BMI (kg/m^2^)	Mean ± SD 29.2 ± 8.2 *N* = 31,605	Mean ± SD 28.7 ± 7.9 *N* = 12,327	Mean ± SD 28.2 ± 7.6 *N* = 12,729	Mean ± SD 28.3 ± 7.7 *N* = 12,209
BMI: < 18.5 k (kg/m^2^)	31,605	777	12,729	796
BMI: 18.50–30 (kg/m^2^)	18,848	7521	7989	7607
BMI: 30–35 (kg/m^2^)	7300	2788	2821	2733
BMI: 35–40 (kg/m^2^)	3726	1348	1375	1337
BMI: > 40 (kg/m^2^)	3674	1126	1152	1144
Neutrophils (#/volume) in blood (10 ∗ 3/μL)	Mean ± SD 360.3 ± 1730.8 *N* = 30,622	Mean ± SD 135.3 ± 1064 *N* = 16,583	Mean ± SD 29.0 ± 473.3 *N* = 14,655	Mean ± SD 30.1 ± 487.6 *N* = 13,805
0–1.5 (10 ∗ 3/μL)	1130	452	526	481
1.50–8 (10 ∗ 3/μL)	19,308	8298	8743	8293
> 8 (10 ∗ 3/μL)	17,577	8499	9166	8572
Hemoglobin A1c	1495	792	1212	1114
< 5.70%	9122	3384	3603	3461
5.70%–6.50%	3228	1294	1407	1328
6.50%–9%	2726	949	933	962
> 9.0%	2525	833	867	851

**Table 6:** Patient demographics for IJV vs SCV analysis between 2014 and 2025.

**Cohort demographics before and after matching** **Comparison 2: Internal jugular vein vs. subclavian vein**				
	**Patients with IJ CVC** **Before matching = 53,530** **After matching = 19,119**		**Patients with SCV CVC** **Before matching = 19,449** **After matching = 19,119**	
**Demographic**	**Before matching**	**After matching**	**Before matching**	**After matching**

Age at index				
Age (in years)	Mean ± SD 61.8 ± 16.3 *N* = 53,530	Mean ± SD 59.5 ± 17.0 *N* = 19,119	Mean ± SD 59.4 ± 18.0 *N* = 19,449	Mean ± SD 59.5 ± 18.0 *N* = 19,119
Sex				
Female	23,398	9317	9353	9127
Male	28,486	9205	9478	9376
Race and ethnicity				
Unknown race	7064	3444	3771	3472
Unknown ethnicity	12,277	5751	6062	5761
White	29,716	11,034	11,022	11,002
Black or African American	9906	3241	3311	3310
Asian	4142	533	513	513
Other race	2093	748	748	738
Hispanic or Latino	5499	1235	1232	1232
Not Hispanic or Latino	35,574	12,133	12,155	12,126

**Table 7:** Patient characteristics for IJV vs SCV analysis between 2014 and 2025.

**Cohort characteristics before and after matching** **Comparison 2: Internal jugular vein vs. subclavian vein**				
	**Patients with IJ CVC** **Before matching = 53,530** **After matching = 19,119**		**Patients with SCV CVC** **Before matching = 19,449** **After matching = 19,119**	
**Characteristic**	**Before matching**	**After matching**	**Before matching**	**After matching**

Diagnoses				
Diabetes mellitus (DM)	14,793	3921	3975	3945
Neoplasms	9026	4563	4664	4403
Chronic kidney disease (CKD)	16,247	2704	2664	2664
Procedure from 7 days prior to same day as index event				
Respiratory ventilation: Less than 24 h	2833	936	956	951
Respiratory ventilation: 24–96 consecutive hours	5538	1804	1827	1822
Respiratory ventilation: Greater than 96 consecutive hours	5553	2151	2153	2134
Introduction of nutritional substance into central vein, percutaneous approach	556	184	183	183
Medications administered from 7 days prior to same day as index event				
Glucocorticoids	20,574	7257	7388	7263
Epinephrine	923	245	268	267
Norepinephrine	11,001	3531	3644	3607
Labs				
Lactate (moles/volume)	Mean ± SD 2.3 ± 2.6 *N* = 16,535	Mean ± SD 2.4 ± 2.6 *N* = 5493	Mean ± SD 2.3 ± 2.5 *N* = 5511	Mean ± SD 2.3 ± 2.5 *N* = 5.498
Lactate: 0–2 mmol/L	12,105	3861	3849	3846
Lactate: 2–4 mmol/L	7540	2697	2643	2632
Lactate: > 4 mmol/L	5023	1706	1678	1671
BMI (kg/m^2^)	Mean ± SD 29.2 ± 8.2 *N* = 31,605	Mean ± SD 28.7 ± 8.0 *N* = 10,516	Mean ± SD 28.4 ± 8.4 *N* = 10,749	Mean ± SD 28.4 ± 8.4 *N* = 10,657
BMI: 0–18.5 k (kg/m^2^)	1672	563	571	568
BMI: 18.50–30 (kg/m^2^)	18,848	6575	6583	6512
BMI: 30–35 (kg/m^2^)	7300	2244	2314	2296
BMI: 35–40 (kg/m^2^)	3726	1001	1024	1024
BMI: > 40 (kg/m^2^)	3666	1088	1121	1117
Neutrophils (#/volume) in blood (10 ∗ 3/μL)	Mean ± SD 360.3 ± 1730 *N* = 30,622	Mean ± SD 138.6 ± 1076 *N* = 9370	Mean ± SD 12.3 ± 133.4 *N* = 9266	Mean ± SD 12.3 ± 133.5 *N* = 9246
0–1.5 (10 ∗ 3/μL)	1130	374	343	343
1.50–8 (10 ∗ 3/μL)	19,308	5922	5897	5881
> 8 (10 ∗ 3/μL)	17,577	5114	5245	5240
Hemoglobin A1c	1495	484	578	572
< 5.70%	9122	2396	2415	2407
5.70%–6.50%	3288	912	907	902
6.50%–9%	2726	723	715	714
> 9.0%	2525	578	585	584

## Data Availability

The data that support the findings of this study are available from TriNetX. Restrictions may apply to the availability of these data, which were used under license for this study. Data are available from https://trinetx.com with the permission of TriNetX.
